# Negative Feedbacks by Isoprenoids on a Mevalonate Kinase Expressed in the *Corpora Allata* of Mosquitoes

**DOI:** 10.1371/journal.pone.0143107

**Published:** 2015-11-13

**Authors:** Pratik Nyati, Crisalejandra Rivera-Perez, Fernando G. Noriega

**Affiliations:** Department of Biological Sciences, Florida International University, Miami, FL, 33199, United States of America; Virginia Tech, UNITED STATES

## Abstract

**Background:**

Juvenile hormones (JH) regulate development and reproductive maturation in insects. JHs are synthesized through the mevalonate pathway (MVAP), an ancient metabolic pathway present in the three domains of life. Mevalonate kinase (MVK) is a key enzyme in the MVAP. MVK catalyzes the synthesis of phosphomevalonate (PM) by transferring the γ-phosphoryl group from ATP to the C_5_ hydroxyl oxygen of mevalonic acid (MA). Despite the importance of MVKs, these enzymes have been poorly characterized in insects.

**Results:**

We functionally characterized an *Aedes aegypti* MVK (*Aa*MVK) expressed in the *corpora allata* (CA) of the mosquito. *Aa*MVK displayed its activity in the presence of metal cofactors. Different nucleotides were used by *Aa*MVK as phosphoryl donors. In the presence of Mg^2+^, the enzyme has higher affinity for MA than ATP. The activity of *Aa*MVK was regulated by feedback inhibition from long-chain isoprenoids, such as geranyl diphosphate (GPP) and farnesyl diphosphate (FPP).

**Conclusions:**

*Aa*MVK exhibited efficient inhibition by GPP and FPP (K*i* less than 1 μM), and none by isopentenyl pyrophosphate (IPP) and dimethyl allyl pyrophosphate (DPPM). These results suggest that GPP and FPP might act as physiological inhibitors in the synthesis of isoprenoids in the CA of mosquitoes. Changing MVK activity can alter the flux of precursors and therefore regulate juvenile hormone biosynthesis.

## Introduction

Juvenile hormones (JH) play a central role in insect development and reproduction [1]. JHs are sesquiterpenoids biosynthesized *de novo* by the *corpora allata* (CA), a pair of endocrine glands connected to the brain [2, 3]. The biosynthetic pathway of JH III involves 13 discrete enzymatic reactions and it is conventionally divided into early (mevalonic acid pathway—MVAP-) and late (JH-branch) steps.

Mevalonate kinase (MVK) is a key enzyme in the MVAP. MVKs (EC 2.7.1.36) are found in the three domains of life as homodimeric proteins [4–7]. They are members of the “GHMP kinase family”, a group of sugar kinases that originally included **g**alactokinases, **h**omoserine kinases, **m**evalonate kinases, and **p**hosphomevalonate kinases [8, 9]. These enzymes catalyze the synthesis of phosphomevalonate (PM) by transferring the γ-phosphoryl group from ATP to the C_5_ hydroxyl oxygen of mevalonic acid (MA) in the presence of a divalent cation [10]. They are involved in the production of cholesterol in mammals [11], as well as JHs in insects. MVK deficiency results in human diseases such as mevalonic aciduria and hyperimmunoglobulinemia D/periodic fever syndrome [12].

The activity of MVK is an important regulatory point in the mevalonate pathway in bacteria [6] and eukaryotes [13]. Previous studies have shown that MVKs are subject to inhibition by MVAP intermediates, such as farnesyl pyrophosphate (FPP) and geranyl pyrophosphate (GPP), which may limit isoprenoid production [13–15]. FPP and GPP exert a competitive inhibition at the ATP binding site of MVK [13, 16]; with greater sensitivity to feedback inhibition in eukaryotic enzymes (K*i* = 34 nM) than bacterial (K*i* = 44 μM) and archaeal (K*i* = 34 μM) enzymes [17, 18]. The diversity of inhibitory mechanisms has permitted the classification of MVKs into three distinct classes. MVK class I are inhibited by metabolites downstream of the diphosphomevalonate carboxylase reaction (IPP, DMAPP, GPP, FPP and longer chain isoprenoids), MVK class II are inhibited by diphosphomevalonate (DPM), but not by metabolites downstream of diphosphomevalonate carboxylase, and MVK class III are not inhibited by isoprenoids [18]. High expression of MVK transcripts has been described in the CA of several insect species, including *A*. *aegypti* [19–21]; however, the catalytic properties of these enzymes have been poorly characterized. A MVK from *Sarcophaga bullata* was partially characterized, showing low affinities for MA and ATP [22].

We characterized a MVK expressed in the CA of the mosquito *Aedes aegypti* (*Aa*MVK). The recombinant *Aa*MVK displays a strong feedback inhibition by long chain isoprenoids, such as geranyl-geranyl pyrophosphate (GGPP), FPP and GPP; with K*i* values of less than 1 μM. The endogenous activity of *Aa*MVK was also strongly inhibited by adding long chain isoprenoids to crude extract of mosquito thoraces (containing the CA). Homology modeling was used to build the structure of *Aa*MVK, which revealed the characteristic GHMP kinase domains, as well as the key amino acids involved in substrate binding and catalytic activity.

## Materials and Methods

### Chemicals

Geranyl-geranyl pyrophosphate (GGPP), farnesyl pyrophosphate (FPP), geranyl pyrophosphate (GPP), isopentenyl pyrophosphate (IPP), dimethyl allyl pyrophosphate (DMAPP) and farnesol (FOL) were purchased from Echelon Biosciences (Salt Lake City, UT). Mevalonic acid (MA), phosphomevalonate (PM), diphosphomevalonate (DPM), phosphoenolpyruvate (PEP) and nicotinamide adenine dinucleotide reduced (NADH) were purchased from Sigma-Aldrich (St. Louis, MO). Pyruvate kinase (PK) and lactate dehydrogenase (LDH) were purchased from LEE biosciences (Maryland Heights, MO).

### Insects

*A*. *aegypti* of the Rockefeller strain were reared at 28°C and 80% relative humidity under a photoperiod of 16 h light: 8 h dark. A cotton pad soaked in 3% sucrose solution was provided to adults.

### Sequence analysis and homology modeling

Sequences similarity searches were performed using the alignment tool BLAST [23]. MVK amino acid sequences were obtained from the National Center of Biotechnology Information and Vector Base. Analyses of degrees of similarity among sequences were performed using the ClustalW tool [24]. *Aa*MV*K* secondary structure was predicted using PDBsum [25]. Amino acid sequence alignments were performed using Muscle [26]. Motifs from aligned sequences were selected, and consensus sequence logos were built using Weblogo [27]. *Aa*MVK tertiary structure was modeled using the protein structure homology-modeling server Swiss v.8.05 and rat MVK (PDB code 1KVK) as template.

### Expression of recombinant A. aegypti mevalonate kinase

The *Aa*MVK cDNA was expressed in *E*. *coli* cells as described by Nyati et al. [28]. Recombinant His-tagged proteins were purified using HiTrap affinity columns and PD-10 desalting columns (Amersham Pharmacia, Piscataway, NJ). Glycerol was added to the enzyme solution (final concentration 50%), and samples were stored at -20°C until used. Protein concentrations were determined using the bicinchoninic acid (BCA) protein assay reagent (Pierce, Rockford, IL). Bovine serum albumin was used as a standard.

### Enzyme assays

The catalytic activity of *Aa*MVK was measured indirectly using a spectrophotometric assay that couples ADP formation to pyruvate synthesis and reduction to lactate [17, 18]. The disappearance of NADH (measured at 340 nm) serves as a measurement for the phosphorylation of MA by MVK. Samples were incubated for 10 min at 30°C. Each 100 μl reaction mixture contained 0.5 mM phosphoenolpyruvate, 0.01 mM DTT, 0.35 mM NADH, 10 mM MgCl_2_, 2 units of LDH, and 2 units of PK in 100 mM Tris-HCl pH 7.6. Phosphorylation of MVA was analyzed in reactions containing ATP (250 μM) and MA (200 μM). Assays were performed in triplicate in 96-well plates (BioTek, Winooski, VT).

Reaction products from the catalytic activity of *Aa*MVK were evaluated by reverse-phase HPLC (RP-HPLC). Briefly, recombinant protein (150 ng) was incubated for 1 h in the reaction buffer (100 mM Tris-HCl pH 7.5, 10 mM MgCl_2_, 0.5 mM DTT) containing: MA (200 μM) and ATP (250 μM). Reactions were terminated by adding 500 μl of acetonitrile, vortexed for 1 min, and centrifuged at 14,000 rpm for 5 min. The organic phase containing PM was recovered, filtered and analyzed by RP-HPLC on a Dionex Summit System (Dionex, Sunnyvale, CA) as previously described [28]. Water and glycerol were used in place of recombinant enzyme as negative controls.

### Kinetic parameters

The Michaelis-Menten constant for MA (*K*_*m-MA*_) was determined at a saturating concentration of ATP (5 mM), with MA concentrations ranging from 0.005 to 2.5 mM. Reactions were initiated with the addition of 150 ng of recombinant *Aa*MVK. The *K*_*m-ATP*_ was determined using saturating concentrations of MA (1.25 mM) and ATP concentrations ranging from 0.005 to 5 mM. The amount of NADH oxidized to NAD^+^ was monitored at 340 nm. To determine steady-state kinetic parameters, data were subjected to nonlinear regression fits to the Michaelis—Menten equation using the GraphPad Prism software (San, Diego, CA).

Inhibition studies were performed in triplicate by adding different MVAP intermediates (DPM, DMAPP, IPP, GPP and FPP) to the reaction mix, as well as GGPP at various concentrations (0–1 μM). Inhibition constants (*K*_*i*_) for GPP, FPP and GGPP were calculated after multi-curve fits using the GraphPad Prism software.

### AaMVK activity in extracts of mosquito thoraces

Mevalonate kinase activities in thoraces of female adult mosquitoes were measured by monitoring the production of PM using RP-HPLC. Thoraces from 24h old 3% sugar-fed females were dissected in *Aedes* physiological saline (APS) (138 mM NaCl, 8.4 mM KCl, 4 mM CaCl_2_, 2 mM MgCl_2_, 12 mM Na_2_HPO_4_ and 42.5 mM sucrose), and transferred to a buffer solution (100 mM Tris-HCl pH 7.5, 10 mM MgCl_2_, 0.01 mM DTT). Thoraces were homogenized for 1 min, sonicated 3 min and centrifuged at 10,000 g for 10 min at 4°C. Supernatants were recovered and used as crude extract (CE) for activity assays as previously described [29]. The protein contents of the CE were measured using the BCA assay. Enzymatic assays were performed using 4 mg of protein as previously described. Boiled crude extract and reactions without enzyme were included as controls. A standard curve was constructed for the quantification of PM.

IPP in CE were measured by RP-HPLC after conversion into the corresponding alcohol by treating samples with 50 μL of 2.5 N HCl for 10 min [30]. Afterward, 500 μL of hexane were added, samples were vortexed for 1 min and centrifuged at 14, 000 rpm for 10 min at 4°C. Organic phases (containing the alcohols) were recovered, filtered through a 0.2 μm nylon filter and quantified by RP-HPLC.

### Statistical analysis

Statistical analyses were performed using the GraphPad Prism Software (San Diego, CA, USA). The results are expressed as means ± S.E.M. Significant differences (P < 0.05) were determined with a one-tailed Student’s t-test or one-way ANOVA followed by a pair-wise comparison of means (Tukey’s test).

## Results

### Molecular characterization of A. aegypti MVK

**Fig 1:**
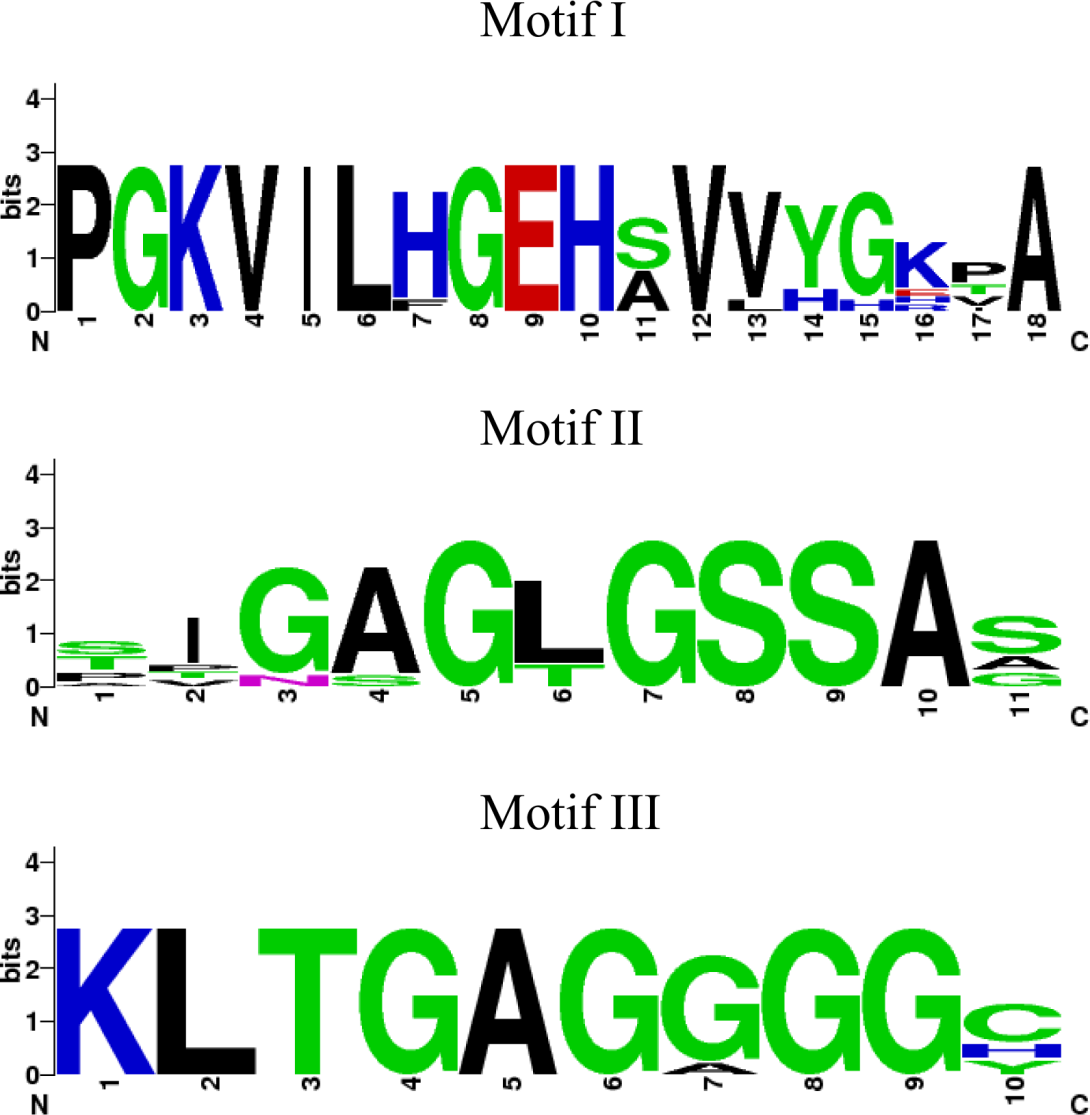
Motifs derived from MVK amino acid sequence alignments display consensus sequences. Sequence logos for motif I, II and II were built using Weblogo [27]. The overall height of the stack indicates the sequence conservation at that position, colors denote the chemical properties of the amino acids. The sequences used are: *Aedes aegypti* (AAEL006435), *Culex quinquefasciatus* (EDS42994), *Anopheles gambiae* (EAA14782), *Drosophila melanogaster* (AGB93455), *Bombyx mori* (NP_001093299), *Danaus plexippus* (EHJ79258), *Apis mellifera* (XP_006558673), *Acyrthosiphon pisum* (XP_001942835) and *Rattus norvegicus* (NP_112325). Colors denote the chemical properties of the amino acids shown in each motif. Polar: G, S, T, Y, C; Neutral: Q; Basic: K, R, H; Acid: D, E; Hydrophobic: A, V, L, I, P, W, F, M.

The full-length *Aa*MVK open reading frame is 1818 bp long (AAEL006435) [31, 32], and encodes a 397-aa protein with a calculated molecular mass of 43.27 kDa and a pI of 5.8. Amino acid sequence alignments of MVKs from insect and vertebrate species revealed 30–44% similarities (S1 Fig). The three conserved motifs that characterize MVKs were highly conserved among all the sequences analyzed, corroborating the functional role of *Aa*MVK as a kinase (Fig 1). Motif I: containing part of the active site (PGKVILXGEHSVVXXXPA); motif II: a conserved glycine-rich motif (SIGXGLGSSAG) that forms a phosphate-binding loop in all GHMP kinases, and motif III: a conserved amino acid sequence (KLTGAGGGGC) that stabilizes the phosphate binding loop [5, 9].

### Structural analysis of the active site of AaMVK

**Fig 2:**
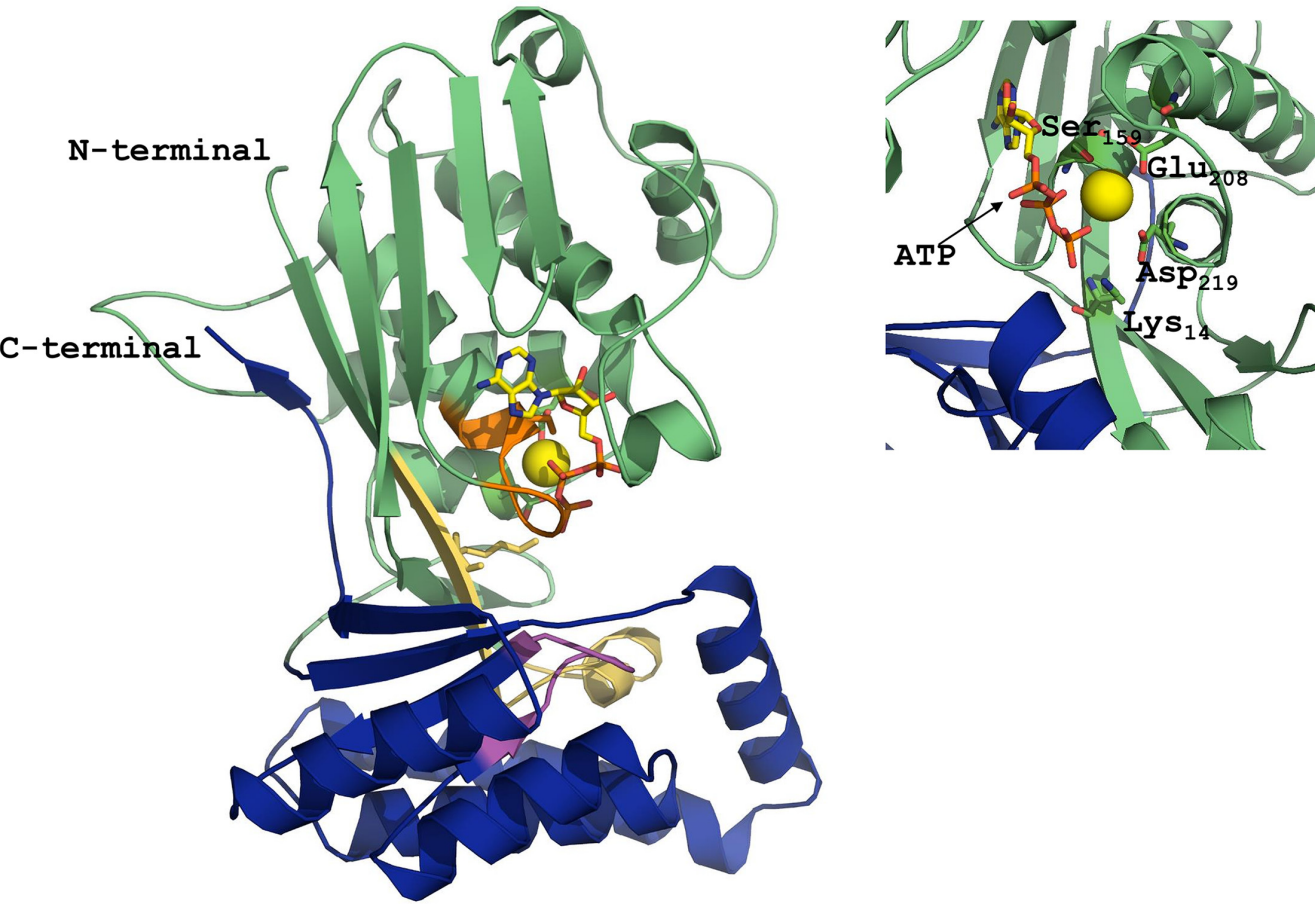
Overall structure of the *A*. *aegypti* MVK in complex with MgATP. The N-terminal domain is shown in green and the C-terminal domain is shown in blue. Motif I (light yellow), Motif II (orange) and Motif III (magenta) are shown in different colors. Key amino acids (Lys_14_, Ser_159_, Glu_208_ and Asp_219_) are shown in the core of the protein. ATP and Mg (yellow ball) are also shown. The model was generated using the rat MVK (PDB code 1KVK) as template, using PyMOL.

The molecular model of *Aa*MVK was built by homology modeling using the rat MVK (PDB: 1kvk), which exhibited 32.7% identity to *Aa*MVK, as template (Fig 2). The analysis of the *Aa*MVK structure revealed a fold consisting of a mixture of α-helices and β-sheets (S2 Fig). The N-terminal domain is composed of ten β sheets and eight α- helices and the C-terminal domain is composed of four helices and two β sheets. The larger N- terminal (include amino acids 1 to 246, 358 to 397) and the smaller C-terminal (include amino acids 247 to 357) domains are arranged in a V-shape that creates a central cleft, with the *Aa*MVK ligand binding pocket, composed by Lys_14_, Ser_159_, Glu_208_, and Asp_219_, located at the cleft between the two domains. Similar structures have been previously described for other MVKs [4, 5, 33].

### Functional characterization of AaMVK

**Fig 3:**
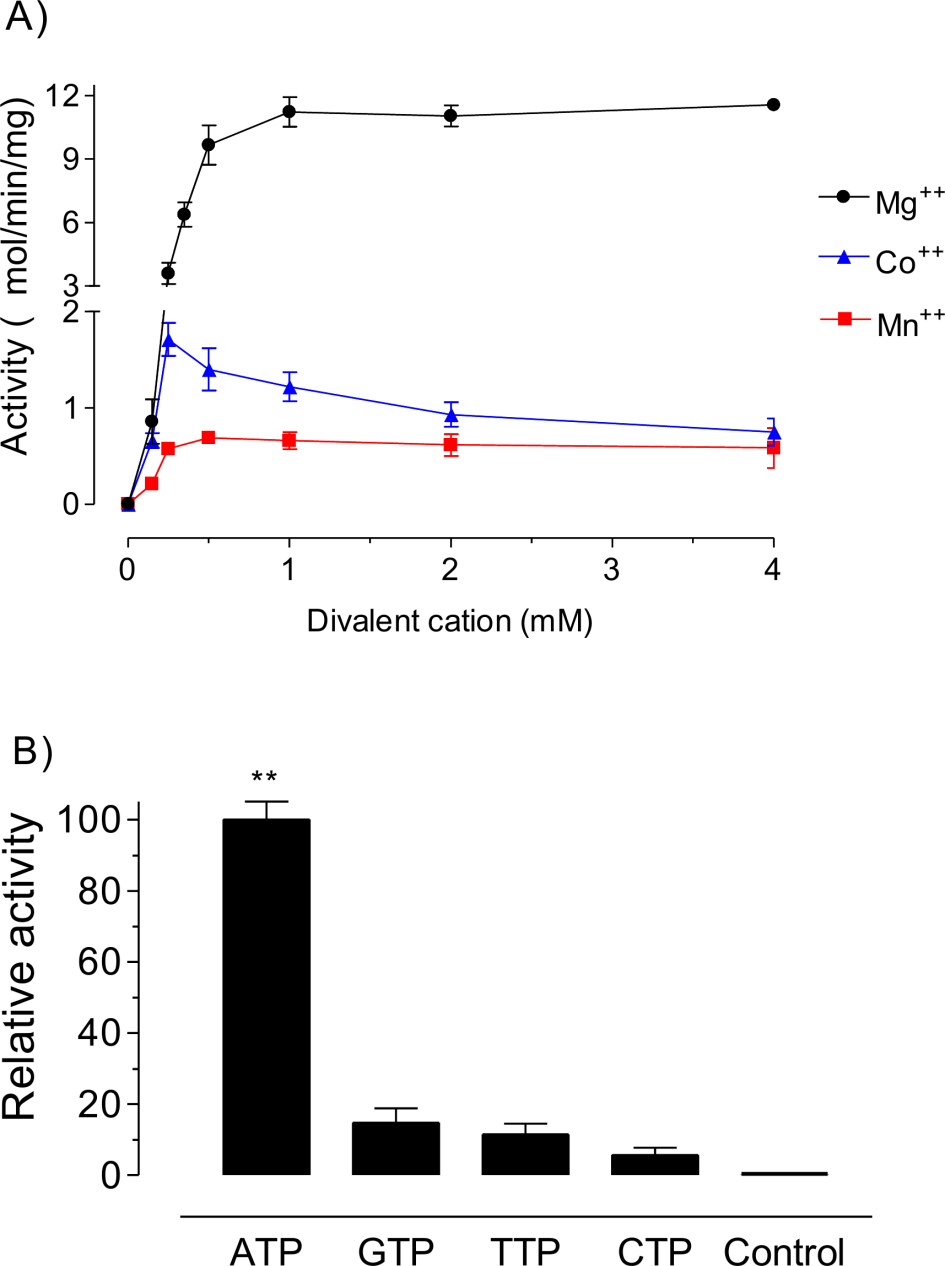
Effect of A) divalent cations and B) specificity of the phosphoryl donor of *Aa*MVK. Kinase activities were measured by the enzyme coupled spectrophotometric assay. A) Metal ion dependence was measured in the presence of ATP (250 μM) and MA (200 μM). Each value represents the means ± S.E. of three replicate assays. B) The specificity of phosphoryl donor were measured in the presence of 10 mM MgCl_2_. Relative activity is defined as a percentage of the highest value recorded (ATP). Each value represents the means ± S.E. of three replicate assays (** P ≤ 0.05).

The recombinant *Aa*MVK was isolated from *E*.*coli* extracts by affinity chromatography, and identified using an anti-His antibody (S3 Fig) as previously described [28]. Optimal catalytic conditions were initially established for *Aa*MVK. The recombinant enzyme displayed activity on a broad range of pH values, with the highest activity at pH 7.5–8.0 (S4 Fig). The catalytic activity of recombinant *Aa*MVK increased in a dose response manner when Mg^2+^ was used as a cofactor. Mn^2+^ and Co^2+^ also enhanced MVK activity to a lesser degree than Mg^2+^ (Fig 3A).

The nucleotide specificity of *Aa*MVK was measured in the presence of different triphosphates phosphoryl donors. Relative rates of MVK activity when ATP, GTP, TTP and CTP were used were 100, 15, 11 and 5 percent respectively (Fig 3B).

### Kinetic analyses of AaMVK

*Aa*MVK showed normal Michaelis-Menten kinetics, which were obtained using a range of ATP concentrations and a fixed mevalonic acid (MA). The apparent K_*m ATP*_ was 140 ± 28 μM. Measurements using a fixed ATP concentration and MA levels that ranged from 0.005 to 2.5 mM indicated that the apparent K_*m MA*_ was 90 ± 18 μM. K_*m*_ values for MA were comparable to those previously described in archaea, bacteria and eukaryotes (Table 1).

**Table 1:** Kinetics parameters of eukaryotic, bacterial and archaeal MVKs.

Species	K_m MA_ (μM)	K_m ATP_ (μM)	V_max_ (μmol min^-1^ mg^-1^)	K_cat_ (s^-1^)	Ki GPP (μM)	Ki FPP (μM)	Reference
*A*. *aegypti*	90	140	33	10.1	0.55	0.44	This Work
*S*. *bullata*	620	4700	ND	ND	ND	ND	[22]
*R*. *norvergicus*	35	950	39	21.9	ND	2.50	[41]
*H*. *sapiens*	150	440	14	ND	0.11	0.10	[13]
*S*. *pneumonia*	236	372	ND	11.0	NI	NI	[18]
*S*. *aureus*	41	339	12	ND	ND	>10	[42]
*M*. *jannaschii*	106	1180	50	28.5	>10	>10	[41]
*M*. *mazei*	68	464	ND	4.3	NI	NI	[18]

ND: Not Determined; NI: not inhibitory.

### Inhibition of AaMVK by long-chain isoprenoids

**Fig 4:**
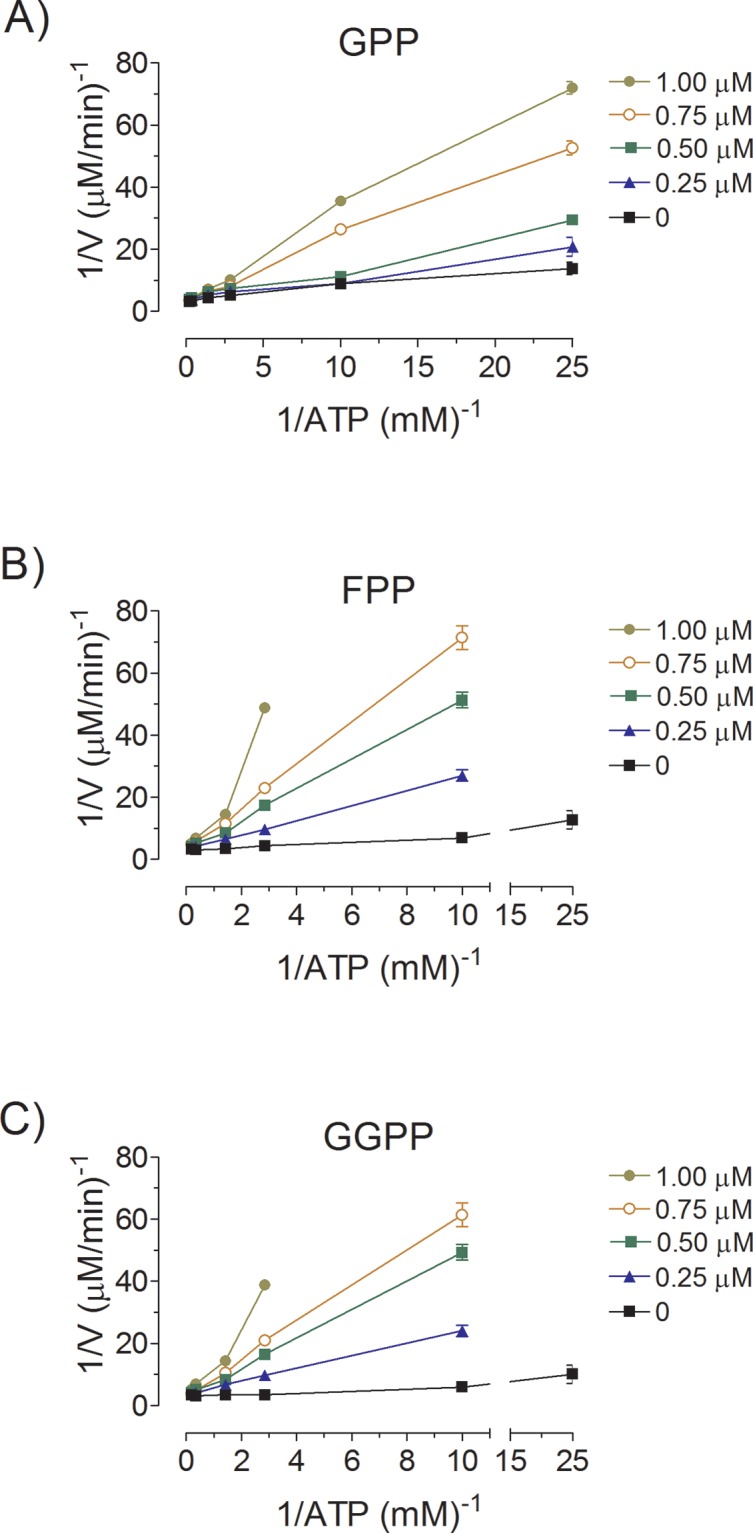
Inhibition of *Aa*MVK activity by GPP (A), FPP (B) and GGPP (C). The rate of MVK activity was measured at different ATP concentrations, without inhibitors and with several fixed concentration of inhibitors (0–1 μM) and MA (200 μM). Secondary plots of slope versus inhibitor concentration indicated that the *Ki* values for GPP, FPP and GGPP were respectively 0.55 ± 0.28 μM, 0.44 ± 0.2 μM and 0.93 ± 0.19 μM.

The sensitivity of *Aa*MVK towards several phosphorylated isoprenoids is shown in Fig 4 and Table 1. *Aa*MVK activity was strongly inhibited by long chain isoprenoids. Our results demonstrated that GGPP, FPP and GPP are competitive inhibitors for the binding of ATP to *Aa*MVK. Their inhibitory capacities (*Ki*) were: GGPP (0.93 ± 0.19 μM), FPP (0.44 ± 0.2 μM) and GPP (0.55 ± 0.28 μM) (S5 Fig). Short chain isoprenoids, such as DMAPP and IPP inhibited only in the micromolar range, with a *K*_*i*_ value greater than 10 μM; while C_6_ compounds, such as PM and DPM, were not inhibitory.

### AaMVK activity in extracts of mosquito thoraces

**Fig 5:**
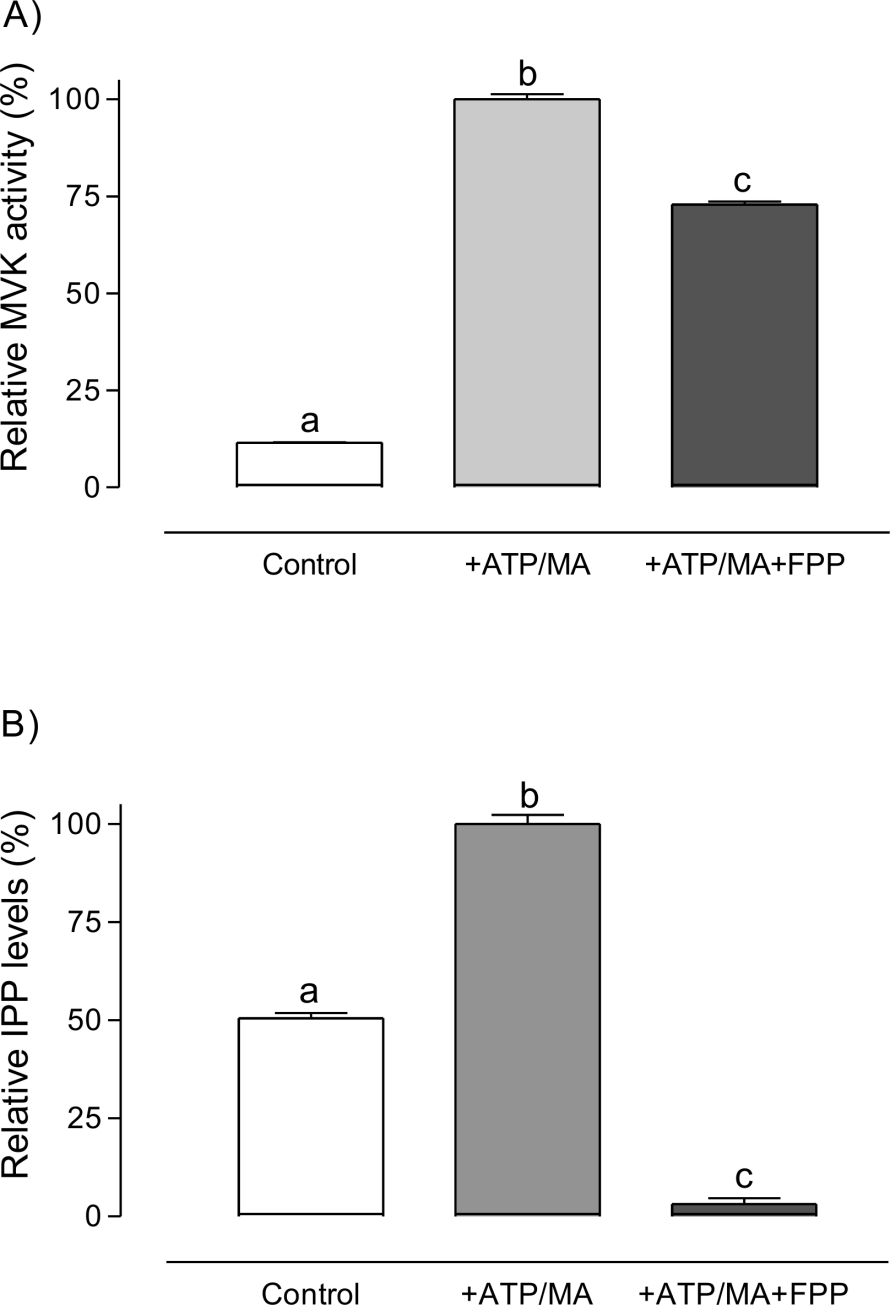
Inhibition of *Aa*MVK activity by FPP in thorax extracts. A) The activity of *Aa*MVK in the absence and presence of FPP was measured by the enzyme-coupled spectrophotometric assay. B) The endogenous levels of IPP derived from the activity of *Aa*MVK in thoraces extracts were measured by RP-HPLC. Optimal reaction conditions were used: ATP (250 μM) and MA (200 μM). Controls did not include substrate or cofactor. Data are expressed as percentage of the highest value recorded. Bars represent the means ± SE of three replicates of extracts from groups of 3 thoraces. Different letters above the columns indicate significant differences among treatments (one way ANOVA p < 0.05, with Tukey’s test of multiple comparisons).

To further assess the inhibitory feedback of isoprenoid on *Aa*MVK, we analyzed the MVK activity in homogenates of mosquito thoraces that contained the CA (24h old sugar-fed females), in the presence of 100 μM FPP. Addition of FPP resulted in a 25% reduction of MVK activity (Fig 5A). When we analyzed the reactions products from the activity of enzymatic extracts from mosquito thoraces on MA, we observed a significant increase in isopentenyl pyrophosphate (IPP) concentration. Since MVK, phosphomevalonate kinase and mevalonate diphosphate decarboxylase share similar reaction conditions [34], these results suggested that in our *in vitro* assay using mosquito homogenates the catalytic transformation of MA into PM continue to generate IPP via diphosphomevalonate (DPM). Changes in the IPP concentration were therefore used as a proxy to study the effect of FPP on MVK activity in mosquito thoraces homogenates. An inhibitory effect of FPP on MVK was confirmed, with the levels of IPP significantly reduced when FPP was added to the extracts, compared to those that were not exposed to FPP (Fig 5B).

## Discussion

The JHs are synthesized through the MVAP, an ancient metabolic pathway present in the three domains of life [7]. The MVAP consists of a main trunk followed by sub-branches that generate a diverse range of essential biomolecules required for cell signaling, membrane integrity, energy homeostasis, protein prenylation and glycosylation [35–38]. Insects lack the cholesterol-synthetic branch present in vertebrates [39], but in the CA the MVAP branches into the synthesis of JH.

Mevalonate kinase is one of three consecutive ATP-dependent enzymes in the MVAP. The primary structure of *Aa*MVK revealed all the characteristic GHMP kinase domains (Pfam: 00288 and Pfam08544), as well as the key amino acids involved in substrate binding and catalytic activity (Lys_14_, Ser_159_, Glu_208_ and Asp_219_) [16, 40]. The structural analysis confirmed that these residues are situated at the active site [5, 17], suggesting that catalysis in insect is also mediated by a base mechanism [5], in which Asp_219_ makes a salt bridge with Lys_14_, with the penta-coordinated γ-phosphate transition state stabilized by Mg^2+^ and the amino acids Glu_208_, Ser_159_ and Lys_14_. The residue Asp_219_ acts as a general base, abstracting a proton from the hydroxyl group of MA, therefore converting MA into an excellent nucleophile that attacks the γ-phosphorus of ATP. The residue Lys_14_ is believed to maintain the aspartate residue in the deprotonated state to facilitate the proton transfer. Several insertions (loops) lying on the surface of the globular structure were identified in the *Aa*MVK. These loops do not contain any catalytic amino acid, but as it has been suggested that might be important conferring protein stability [5]. Although disulfide bridges confer thermostability to prokaryote MVKs [41], the absence of disulfide bridges is another important feature that the *Aa*MVK shares with other eukaryotic MVKs.

Like many ATP-dependent reactions, *Aa*MVK requires divalent metal cations for catalysis. The function of the divalent metal cation is to anchor the diphosphate moieties and to facilitate ionization of allylic substrates [18]. Although in insects it seems that the essential cation *in vivo* is Mg^2+^, our results shown that *Aa*MVK can replace Mg^2+^ *in vitro* by other divalent cations such as Mn^2+^ and Co^2+^. Similarly, in the process of phosphorylation, although other nucleotide triphosphates including GTP, CTP and TTP can partially substitute for ATP as phosphoryl donors *in vitro*, most likely ATP is the preferential *in vivo* phosphoryl donor. The results for the analysis of cofactor requirements, phosphoryl source and optimal pH of *Aa*MVK were in agreement with those described for previously characterized MVKs [42, 43]. Our kinetic studies revealed that the V_max_ for the formation of PM in mosquitoes was comparable to that described for other MVK’s, ranging from 12 to 50 μmol min^-1^ mg^-1^ (Table 1). The *Aa*MVK Michaelis-Menten constants for mevalonate (*Km*_*MA*_) and ATP (*Km*_*ATP*_) were in the range of those previously described for other MVKs (Table 1), which also have higher affinity for MA than ATP. Comparisons of kinetics between purified and recombinant enzymes are not always straightforward; much more difficult is to compare their activities with those of crude extracts. Discrepancies between the kinetic properties of purified and recombinant enzymes from the same species have been reported for other MVP enzymes [44]. Conclusive evidence linking the activity of the recombinant *Aa*MVK with the activity detected in extracts is missing; but the fact that *Aa*MVK is a highly conserved protein, encoded by a single annotated gene in the *A*. *aegypti* genome, and with transcripts enriched in the CA [20], suggest that both activities correspond to the same protein.

*Aa*MVK mRNA expression levels in the CA are concurrent with JH biosynthesis titers in female mosquitoes [20, 31]. MVK transcripts in *Bombyx mori* also correlate with JH synthesis [19], suggesting an important role of this enzyme in the regulation of the JH pathway. The mevalonate pathway is subject to multivalent transcriptional and post-transcriptional regulation, primarily at the level of HMG-CoA reductase [35]; however, it is becoming increasingly clear that regulation of MVK catalysis plays also an important modulatory role. A regulatory mechanism for controlling MVK activity is feedback inhibition by the presence of isoprenoids [17, 42, 45]. The competitive inhibition results from the interaction of the isoprenoid binding site of the phosphoryl group of ATP [17].

*Aa*MVK is a class I enzyme, exhibiting efficient inhibition by GPP and FPP (Ki less than 1 μM), and none by IPP and DPPM. It is interesting that the two products of a single enzyme (FPP synthase) are specific in their inhibition of *Aa*MVK. The possibility that GPP and FPP act as physiological inhibitors (*in vivo*) in the synthesis of JH in mosquito is strengthened when considering the inhibition exerted by these two metabolites on the MVK activity present in crude extracts of female mosquito thoraces containing the CA. However, further analysis are required to evaluate their significance as regulators *in vivo*.

The activity of *Aa*MVK in the CA of female mosquito shows dramatic changes during the gonotrophic cycle that correlate well with changes in JH biosynthesis [34]. *Aa*MVK activity is very low in newly emerged adult females (30 fmol/CA/h), it increases more than 200 folds by 12 h after adult eclosion (4500 fmol/CA/h), and markedly decreases with the decline of JH synthesis by 24 h after blood feeding (20 fmol/CA/h) [34]. Although rate limiting bottlenecks have been proposed at single specific steps in both the MVAP and JH-branch in the CA of different insects, our previous studies suggested that there are multiple regulatory points and they change in different physiological stages [34, 46]. Further studies will be necessary to reveal if *Aa*MVK plays a key role restricting the flux into JH III at specific physiological conditions.

## Supporting Information

S1 Fig Amino acid sequence alignment of selected mevalonate kinases. Accession numbers: *Rattus norvegicus* (NP_112325), *Mus musculus* (AAF00700), *Bos taurus* (NP_001015528), *Homo sapiens* (AAB59362), *Danio rerio* (NP_001007350), *Apis mellifera* (XP_006558673), *Bombyx mori* (NP_001093299), *Danaus plexippus* (EHJ79258), *Drosophila melanogaster* (AGB93455), *Anopheles gambiae* (EAA14782), *Aedes aegypti* (AAEL006435), *Culex quinquefasciatus* (EDS42994), *Acyrthosiphon pisum* (XP_001942835), *Arabidopsis thaliana* (AED93690), *Panax notoginseng* (AFN02124), *Streptococcus pneumoniae* PNI0360 (ELU87568), *Staphylococcus aureus* (ABR51486), *Bacillus coagulans* (AEP00201). The three conserved GHMP motifs are highlighted in grey. Insertions not involved in the catalytic function of MVKs are underlined. Residues involved in binding of the phosphate are marked in red. Below the protein sequences is a key denoting conserved sequence (*), conservative (:) and semi-conservative (.) modifications.
(TIF)

S2 Fig Topology model of *Aa*MVK build using rat MVK (PDB: 1KVK) as template. Alpha helix (helices) and beta sheets (arrows) are shown along the amino acid sequence. Beta turns (β), gamma turns (γ) and beta hairpins (red curved lines) are also indicated. Motifs I—III are boxed. Amino acids involved in catalysis are indicated with black arrows.
(TIF)

S3 Fig Recombinant *Aa*MVK : Analysis of the purified recombinant *Aa*MVK by (A) SDS PAGE and (B) Western blot probed with an anti-His tag antibody. Lane contents of the gel were: 1, molecular weight standard; 2 and 3, two concentrations of purified recombinant *Aa*MVK (5 and 15μg). Molecular weights of protein standards are depicted on the Y axis.
(TIF)

S4 Fig pH curve. The *Aa*MVK activity was investigated at different pHs using mevalonic acid as substrate (200 μM) in the presence of 10 mM MgCl_2_. Two different buffers were used to generate the pH gradient: MES at pH 5.5 to 7 and Tris-HCl at pH 7 to 9. The optimum pH was found to be 7.5 to 8.0; with the enzyme exhibiting 60–70% of its optimum activity over a rather broad pH range (7 to 8.5). Activities are expressed as μmol of product produced by min per mg of enzyme. Each value represents the means ± S.E. of three replicate assays.
(DOCX)

S5 Fig Plot of the slopes obtained from the Fig 4 versus the inhibitor concentration (0–1 μM). A) GPP, B) FPP and C) GGPP.
(DOCX)
