# Supplementary figures and images for: Negative Feedbacks by Isoprenoids on a Mevalonate Kinase Expressed in the Corpora Allata of Mosquitoes
Source: PLoS One. 2015 Nov 13;10(11):e0143107. doi: 10.1371/journal.pone.0143107 (PMC4643977; doi:10.1371/journal.pone.0143107)

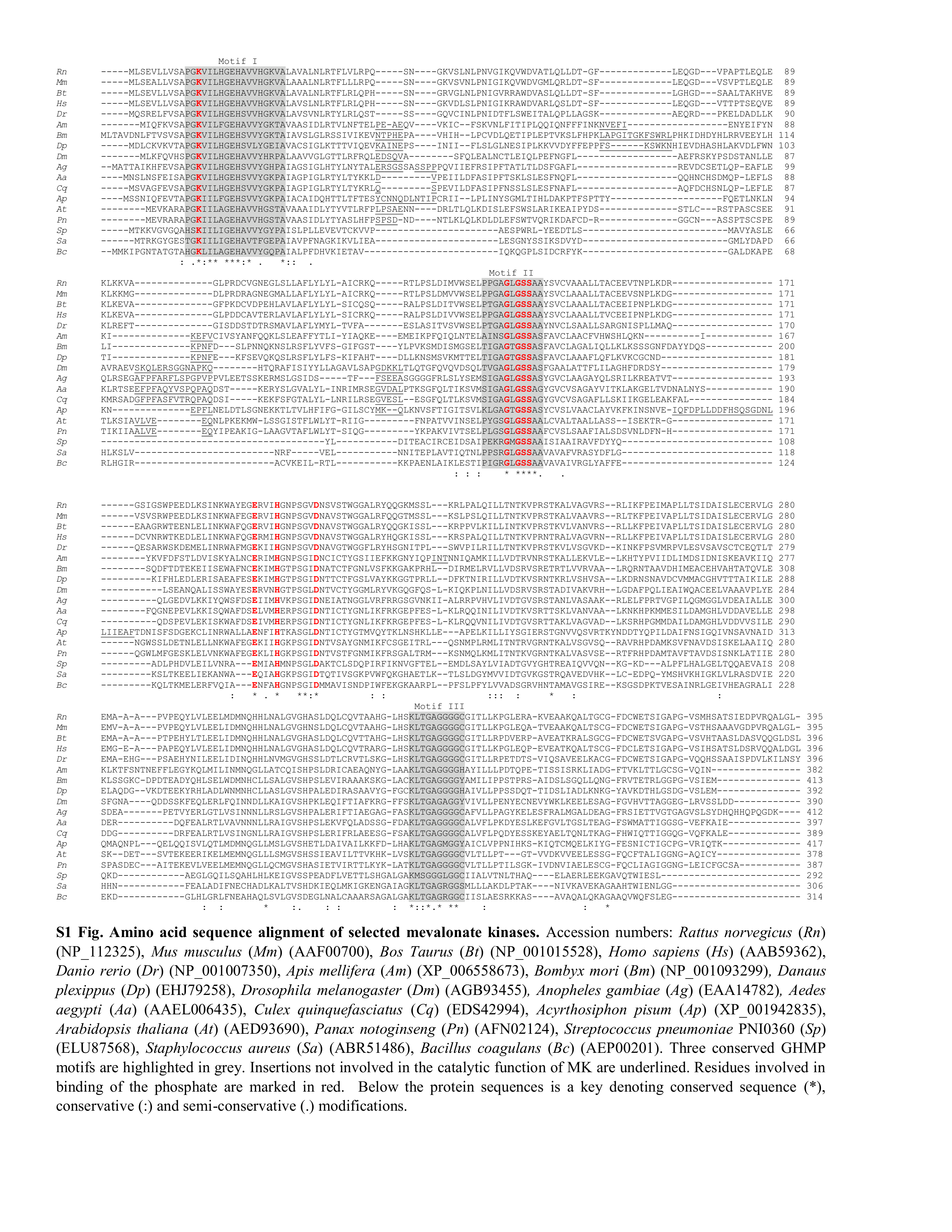

Supplement: S1 Fig — Accession numbers: Rattus norvegicus (NP_112325), Mus musculus (AAF00700), Bos taurus (NP_001015528), Homo sapiens (AAB59362), Danio rerio (NP_001007350), Apis mellifera (XP_006558673), Bombyx mori (NP_001093299), Danaus plexippus (EHJ79258), Drosophila melanogaster (AGB93455), Anopheles gambiae (EAA14782), Aedes aegypti (AAEL006435), Culex quinquefasciatus (EDS42994), Acyrthosiphon pisum (XP_001942835), Arabidopsis thaliana (AED93690), Panax notoginseng (AFN02124), Streptococcus pneumoniae PNI0360 (ELU87568), Staphylococcus aureus (ABR51486), Bacillus coagulans (AEP00201). The three conserved GHMP motifs are highlighted in grey. Insertions not involved in the catalytic function of MVKs are underlined. Residues involved in binding of the phosphate are marked in red. Below the protein sequences is a key denoting conserved sequence (*), conservative (:) and semi-conservative (.) modifications. (TIF) [file pone.0143107.s001.tif]

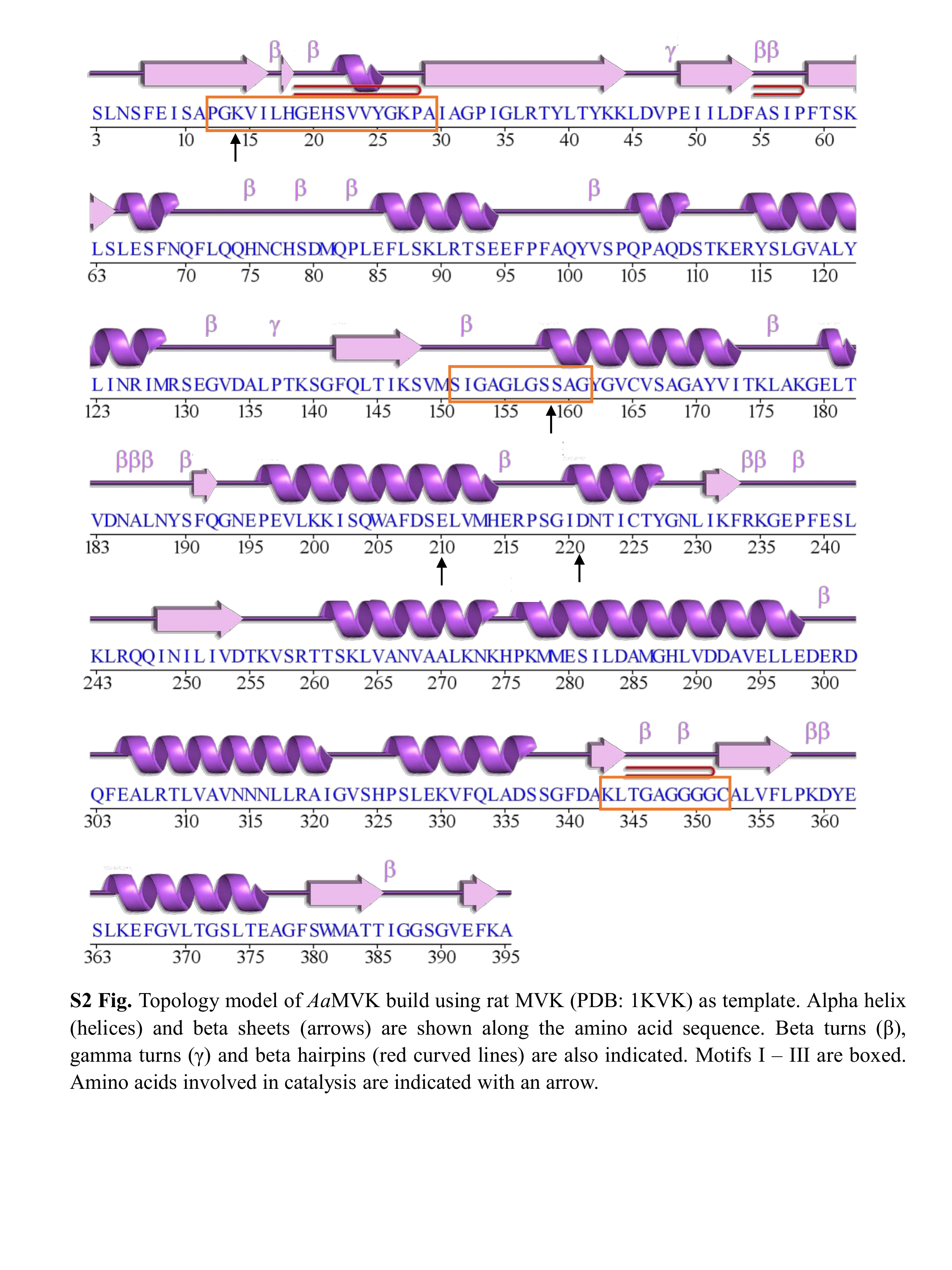

Supplement: S2 Fig — Alpha helix (helices) and beta sheets (arrows) are shown along the amino acid sequence. Beta turns (β), gamma turns (γ) and beta hairpins (red curved lines) are also indicated. Motifs I—III are boxed. Amino acids involved in catalysis are indicated with black arrows. (TIF) [file pone.0143107.s002.tif]

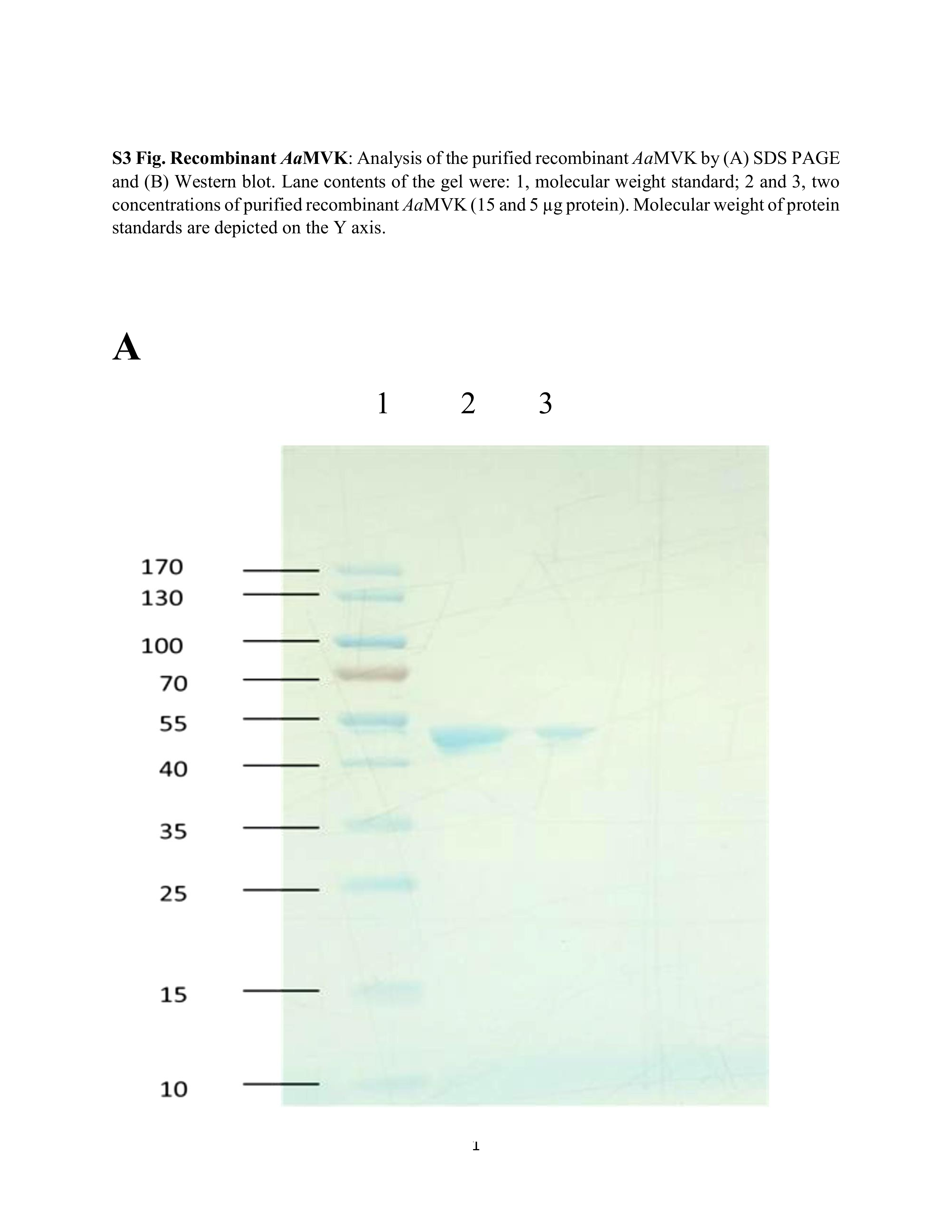

Supplement: S3 Fig — : Analysis of the purified recombinant AaMVK by (A) SDS PAGE and (B) Western blot probed with an anti-His tag antibody. Lane contents of the gel were: 1, molecular weight standard; 2 and 3, two concentrations of purified recombinant AaMVK (5 and 15μg). Molecular weights of protein standards are depicted on the Y axis. (TIF) [file pone.0143107.s003.tif]
