# Supplementary material for: Negative Feedbacks by Isoprenoids on a Mevalonate Kinase Expressed in the Corpora Allata of Mosquitoes
Source: PLoS One. 2015 Nov 13;10(11):e0143107. doi: 10.1371/journal.pone.0143107 (PMC4643977; doi:10.1371/journal.pone.0143107)
Supplement: S4 Fig — The AaMVK activity was investigated at different pHs using mevalonic acid as substrate (200 μM) in the presence of 10 mM MgCl2. Two different buffers were used to generate the pH gradient: MES at pH 5.5 to 7 and Tris-HCl at pH 7 to 9. The optimum pH was found to be 7.5 to 8.0; with the enzyme exhibiting 60–70% of its optimum activity over a rather broad pH range (7 to 8.5). Activities are expressed as μmol of product produced by min per mg of enzyme. Each value represents the means ± S.E. of three replicate assays. (DOCX) [file pone.0143107.s004.docx]

**S4 Fig. pH curve**. The *Aa*MVK activity was investigated at different pHs using mevalonic acid as substrate (200 µM) in the presence of 10 mM MgCl_2_. Two different buffers were used to generate the pH gradient: MES at pH 5.5 to 7 and Tris-HCl at pH 7 to 9. The optimum pH was found to be 7.5 to 8.0; with the enzyme exhibiting 60-70% of its optimum activity over a rather broad pH range (7 to 8.5). Activities are expressed as µmol of product produced by min per mg of enzyme. Each value represents the means ± S.E. of three replicate assays.
