# Supplementary material for: Negative Feedbacks by Isoprenoids on a Mevalonate Kinase Expressed in the Corpora Allata of Mosquitoes
Source: PLoS One. 2015 Nov 13;10(11):e0143107. doi: 10.1371/journal.pone.0143107 (PMC4643977; doi:10.1371/journal.pone.0143107)
Supplement: S5 Fig — A) GPP, B) FPP and C) GGPP. (DOCX) [file pone.0143107.s005.docx]

**S5 Fig. Relationship between the slopes obtained from the Fig. 4 versus the inhibitor concentrations (0 – 1 µM).** A) GPP; B) FPP and C) GGPP.
